# Can hippocampal subfield measures supply information that could be used to improve the diagnosis of Alzheimer’s disease?

**DOI:** 10.1371/journal.pone.0275233

**Published:** 2022-11-03

**Authors:** Balaji Kannappan, Jan te Nijenhuis, Yu Yong Choi, Jang Jae Lee, Kyu Yeong Choi, Irena Balzekas, Ho Yub Jung, Youngshik Choe, Min Kyung Song, Ji Yeon Chung, Jung-Min Ha, Seong-Min Choi, Hoowon Kim, Byeong C. Kim, Hang Joon Jo, Kun Ho Lee

**Affiliations:** 1 Gwangju Alzheimer’s & Related Dementias Cohort Research Center, Chosun University, Gwangju, South Korea; 2 Department of Biomedical Science, Chosun University, Gwangju, South Korea; 3 Department of Neurology, Mayo Clinic, Rochester, Minnesota; 4 Department of Computer Engineering, Chosun University, Gwangju, South Korea; 5 Korea Brain Research Institute, Daegu, South Korea; 6 Department of Neurology, Chonnam National University Medical School and Hospital, Gwangju, South Korea; 7 Department of Neurology, Chosun University Hospital, Gwangju, South Korea; 8 Department of Nuclear Medicine, Chosun University Hospital, Gwangju, South Korea; 9 Department of Neurology, Chonnam National University Medical School, Gwangju, South Korea; 10 Department of Physiology, College of Medicine, Hanyang University, Seoul, South Korea; Nathan S Kline Institute, UNITED STATES

## Abstract

The diagnosis of Alzheimer’s disease (AD) needs to be improved. We investigated if hippocampal subfield volume measured by structural imaging, could supply information, so that the diagnosis of AD could be improved. In this study, subjects were classified based on clinical, neuropsychological, and amyloid positivity or negativity using PET scans. Data from 478 elderly Korean subjects grouped as cognitively unimpaired β-amyloid-negative (NC), cognitively unimpaired β-amyloid-positive (aAD), mild cognitively impaired β-amyloid-positive (pAD), mild cognitively impaired—specific variations not due to dementia β-amyloid-negative (CIND), severe cognitive impairment β-amyloid-positive (ADD+) and severe cognitive impairment β-amyloid-negative (ADD-) were used. NC and aAD groups did not show significant volume differences in any subfields. The CIND did not show significant volume differences when compared with either the NC or the aAD (except L-HATA). However, pAD showed significant volume differences in Sub, PrS, ML, Tail, GCMLDG, CA1, CA4, HATA, and CA3 when compared with the NC and aAD. The pAD group also showed significant differences in the hippocampal tail, CA1, CA4, molecular layer, granule cells/molecular layer/dentate gyrus, and CA3 when compared with the CIND group. The ADD- group had significantly larger volumes than the ADD+ group in the bilateral tail, SUB, PrS, and left ML. The results suggest that early amyloid depositions in cognitive normal stages are not accompanied by significant bilateral subfield volume atrophy. There might be intense and accelerated subfield volume atrophy in the later stages associated with the cognitive impairment in the pAD stage, which subsequently could drive the progression to AD dementia. Early subfield volume atrophy associated with the β-amyloid burden may be characterized by more symmetrical atrophy in CA regions than in other subfields. We conclude that the hippocampal subfield volumetric differences from structural imaging show promise for improving the diagnosis of Alzheimer’s disease.

## Introduction

Alzheimer’s disease (AD) is a non-curable and irreversible neurodegenerative disease that accounts for approximately 70% of all dementia cases. In 2015, around 46.8 million people worldwide were living with dementia, which is estimated to rise to approximately 131.5 million by 2050. In addition, the estimated cost of the disease is about a trillion US dollars in 2018 and is expected to double by 2030 [1]. AD is histopathologically characterized by neuritic plaques composed of amyloid-beta (Aβ) protein aggregates and neurofibrillary tangles composed of tau protein [2, 3]. There are a few criteria that can be combined to diagnose AD: 1) Aβ depositions quantified using cerebrospinal fluid analysis or *in vivo* amyloid positron emission tomography (PET) imaging, 2) anatomical changes in the brain detected through structural magnetic resonance imaging (MRI), and 3) cognitive impairments assessed through neuropsychological tests [4].

The first AD-related change is the Aβ accumulation, which is believed to start at least two decades before any other clinical symptoms [5, 6], and the testing for amyloids using PET imaging comes with various problems. First, amyloid imaging of both asymptomatic subjects and subjects with cognitive complaints unconfirmed after a clinical examination is considered inappropriate and also practically highly challenging [7]. Second, approximately 10–30% of cognitively normal controls test amyloid positive by PET imaging [8–10], and third, the high costs are currently not covered by Medicare or other insurances. So, it will be helpful to have a reliable additional parameter or complementary information that can help to improve the AD diagnosis and to justify the use of PET imaging in certain unclear or questionable diagnoses. In search of this complementary information, we investigated the second AD-related change which is hippocampal volume atrophy, often examined through structural MRI.

Quantitative analysis of structural MRI can serve as an *in vivo* surrogate for the severity of disease in various stages of disease progression [11–14]. Hippocampal volumetry is among the highly discussed and studied quantitative MRI measures and is considered a powerful non-invasive biomarker for AD in diagnostic criteria and clinical trials [15–18]. The hippocampus is a non-homogeneous structure with histologically distinct subfields. Each subfield is believed to be functionally distinct, performing functions related to learning and memory, certain aspects of motor control, regulation of emotional behavior, and regulation of hypothalamic functions, among others (S1 Table) [19]. However, traditionally, the limitations of MRI resolution and the lack of consistent and reliable segmentation methods forced researchers to consider the hippocampus as a single homogeneous structure [20–22]. Recent advances in segmentation techniques made it possible to process large subject groups and automatically segment the hippocampus into its various subfields more efficiently, swiftly, and with greater reproducibility [23].

However, recent research [24] suggests that automated segmentation techniques must be employed with caution on the relatively low-resolution MRI data currently available. Despite these limitations, we believe that dividing the hippocampus into parts or subfields might provide extra information compared to a focus on the total hippocampus. An example of the information that the subregions can provide is the pattern of neuronal loss in the CA1 of the hippocampus, which is qualitatively different and higher in AD subjects than in normal aging subjects [25, 26]; another example is that of the DG, where subject groups with various neuropsychological disorders and high levels of stress have shown more severe adverse effects than controls, suggesting that these impacts act selectively on the subfields and not on the whole hippocampus [27]. So, the localization of subfield-specific volume loss or neurodegeneration could thus be a harbinger of future damage. Additionally, knowledge about the possible differences in the hippocampal subfields between the different diagnostic groups will encourage the development of newer accurate segmentation protocols.

The number of studies on changes in hippocampal subfield volume associated with the Aβ burden and cognitive status in AD and stages preceding AD is limited. In this study, we tried to improve the diagnosis of AD by not taking the traditional focus on the total hippocampus but instead focusing on hippocampal subfield volume. We expected 1) asymmetry in the subfield volumes between the groups, 2) larger subfield volumes in the Aβ negative groups than in the Aβ positive groups, and 3) reduction in the subfield volumes along the AD continuum. When most of the findings are in the expected directions, the information might be used to improve the diagnosis of Alzheimer’s disease.

## Methods

### Study participants

The regional ethics committee approved the study, and written consent was obtained from the participants, family members, or caregivers. Subjects were first classified into three groups—normal control (NC), mildly cognitively impaired (MCI), and Alzheimer’s disease dementia (ADD). The clinical diagnosis of probable AD was made according to the National Institute of Neurological and Communicative Diseases and Stroke/Alzheimer’s Disease and Related Disorders Association Alzheimer’s criteria [4]. Controls had no evidence of neurological disease or impairment in cognitive function or activities of daily living. Individuals with a focal lesion on the brain identified using MRI (magnetic resonance imaging), a history of head trauma, or a psychiatric disorder that could affect mental function were excluded. The diagnosis of MCI was made according to the National Institute on Aging-Alzheimer’s Association’s (NIA-AA) criteria [28]. Depending on the PET scans (positive or negative) and as summarized in Table 1, (S2 Table and S2 Fig), 478 study subjects (NC: n = 192; β-amyloid negative cognitively unimpaired [NC-]), asymptomatic AD (n = 34; β-amyloid positive cognitively unimpaired [aAD or NC+]), cognitive impairments that are not dementia (CIND) (n = 118; β-amyloid negative mildly cognitively impaired [MCI-]), prodromal AD (n = 34; β-amyloid positive mildly cognitively impaired [pAD or MCI+]), Alzheimer’s disease dementia negative (n = 30; β-amyloid negative severely cognitively impaired [ADD-]), and Alzheimer’s disease dementia (n = 70; β-amyloid positive severely cognitively impaired [ADD+]) were recruited by the Gwangju Alzheimer’s disease and Related Dementias (GARD) cohort research center at Chosun University in Gwangju, Republic of Korea.

**Table 1:** Demographic characteristics of the study population.

	NC	aAD	CIND	pAD	ADD-	ADD+
Number of subjects (n)	192	34	118	34	30	70
Age^a,b^	71.95±5.30	73.80±4.21	72.50±6.96	74.17±6.11	73.76±6.48	72.18±6.92
Male (%)^c^	46.35	47.05	43.22	67.64	50	48.57
Level of education (years)^a,d^	9.32±5.50	8.79±5.75	9.66±5.15	8.82±4.93	9.13±6.02	7.48±5.03
MMSE^a,e^	27.43±2.03	27.41±2.09	25.17±3.39	25.23±3.14	19.06±7.86	19.41±5.73

Values are expressed as mean ± standard deviation (SD).

NC, normal controls; ADD, Alzheimer’s disease dementia; aAD, asymptomatic Alzheimer disease; pAD, prodromal Alzheimer disease; CIND, cognitive impairments that are not dementia; MMSE, Mini Mental State Examination.

^a^The P-values were calculated using general linear model; Bonferroni post hoc test was also performed when F-test was significant.

^b^Main interaction among groups: F_5, 478_ = 1.29, p = 0.26. *(Age)*

^c^The P-value were calculated using the χ^2^ test: χ^2^ = 6.60, p = 0.25. *(Gender)*

^d^Main interaction among groups: F_5,478_ = 2.54, p = 0.02. Post hoc: CIND versus ADD+, 0.01, others were insignificant. *(Education)*

^e^Main interaction among groups: F_5, 478_ = 73.44, p = 1.08E-56. post hoc: NC versus aAD, 1.00; NC versus pAD, 8.99E-3; NC versus ADD+, 5.77E-45; NC versus CIND, 3.46E-7; NC versus ADD-, 1.61E-29; aAD versus pAD, 0.04; aAD versus ADD+, 2.85E-24; aAD versus CIND, 2.58E-3; aAD versus ADD-, 2.29E-20; pAD versus ADD+, 1.90E-12; pAD versus CIND, 1.00; pAD versus ADD-, 5.93E-11; CIND versus ADD+, 8.71E-22; CIND versus ADD-, 1.14E-15; ADD+ versus ADD-, 1.00. *(MMSE)*

### Clinical and neuropsychological assessments

Participants underwent testing using the Seoul Neuropsychological Screening Battery, a comprehensive neuropsychological battery of tests and subtests assessing five cognitive domains: memory (subtests: Orientation and Verbal and Visual Memory), language (Korean version of the Boston Naming Test and written Calculation Trails), attention (Forward and Backward Digit Span), visuospatial functions (Copying Test from the Rey Complex Figure Test), and frontal/executive functions (Motor Impersistence, Contrasting Program, Go-no-go Test, Fist-edge-palm task, and the Luria Loop task) [29, 30]. In addition, the battery of tests included the Korean version of the Mini-Mental State Examination (MMSE) [31], the Clinical Dementia Rating [32], and the Seoul Instrumental Activities of Daily Living [33]. Control subjects did not show any evidence of neurological disease or impairment in cognitive function or activities of daily living.

### MRI acquisition

Contiguous 0.8 mm sagittal Magnetization-Prepared Rapid Acquisition Gradient Echo (MPRAGE) images of the whole brain examined at the GARD cohort research center was acquired using a 3T MR scanner (Skyra, Siemens) with the following parameters: TR = 2300 ms; TE = 2.143 ms; TI = 900 ms; 9 flip angles; FoV = 256x256; matrix = 320x320; number of slices = 178.

### MRI data processing

High-resolution structural T1-weighted images were processed using the FreeSurfer software package (v5.3.0 & v6.0.0; Athinoula A. Martinos Center for Biomedical Imaging, Harvard University, Cambridge, MA, USA) on a Linux environment using a 64-bit CentOS 7 operating system. The complete documentation of the FreeSurfer pipeline and methodologies can be found elsewhere [34–38]. Complete automated processing, including cortical and subcortical labeling using the Desikan–Killiany atlas, was performed on each subject. Then, the hippocampal subfields were accessed using FreeSurfer v6.0.0, sub-dividing the hippocampus into subfields, namely the hippocampal tail (Tail), Sub, CA1, hippocampal fissure (fissure), presubiculum (PrS), parasubiculum (PaS), molecular layer (ML), granule cells/molecular layer/dentate gyrus (GCMLDG), CA3, CA4, fimbria, and hippocampal-amygdala transition area (HATA) (S1 Fig) [23]. The hippocampal subfields segmentation was performed for all groups using only FreeSurfer (v6.0.0). It should be noted that the CA3 sector includes the CA2 sector in the hippocampal subfield atlas used. In the current study, we excluded the whole hippocampus volumes from all analyses, as the primary focus was on the subfields.

### PET imaging

Subjects underwent a PET scan 90 min after intravenous injection of 300 MBq ^18^F-florbetaben using a dedicated Discovery ST PET-CT scanner (General Electric Medical Systems, Milwaukee, WI, USA). Non-contrast-enhanced computed tomography (CT) scans were used for attenuation correction with technical parameters of 120 Kvp, 10–130 mAs, eight slices, helical, and 3.79 mm slice thickness. PET and CT scan data were reconstructed using ordered subset expectation maximization after attenuation correction with two iterations and 21 subsets. A Gaussian filter was applied with 5.14 mm FWHM to reconstruct a 128 × 128 matrix with 3.27-mm slice thickness.

### PET image data processing

PET images were assessed according to a predefined regional cortical tracer uptake (RCTU) scoring system (“1” = no uptake, “2” = minor uptake, “3” = pronounced uptake) for four brain regions (frontal cortex, posterior cingulate, lateral temporal cortex, parietal cortex). Details of a three-grade scoring system using RCTU scores for the amyloid plaque load have been provided previously [39]. For the PiB-PET images, the mean retention value of the global cortical region of interest (ROI) was used to define the global cerebral Aβ deposition as amyloid-positive if the mean standard value uptake ratio was > 1.4 in at least one of the ROIs, including the frontal, lateral parietal, lateral temporal, or posterior cingulate-precuneus [40].

### Statistical analyses

All statistical analyses were performed using IBM SPSS Statistics (Version 23.0., Armonk, NY: IBM Corp.) All analyses were two-tailed and controlled for the covariates: age, sex, years of education, and estimated total intracranial volume. One-way analysis of variance and Bonferroni post hoc correction for multiple comparisons was used for continuous demographic variables, and chi-squared tests were performed for categorical demographic variables. A series of analysis of covariance (ANCOVA) on the estimated subfield volumes adjusting for the covariates were performed comparing the six diagnosis groups. The P-values of the comparisons between the diagnosis groups were corrected using the Bonferroni correction for multiple comparisons. The Bonferroni correction was performed across the 24 regions and six groups (Table 2). Further post hoc analysis of the subfields that met the minimum standards of significance after the Bonferroni correction for multiple comparisons were performed for pairwise comparisons between the two groups. Here, we considered p < 0.05 as significant (Table 3). The left-right hemispheric volumetric asymmetry comparisons were evaluated using the *t-test* (Table 4). p < 0.05 was considered statistically significant. Additionally, we investigated the hemispheric volumetric correlation among the six diagnostic groups using Pearson’s correlation, and again we used a statistical significance of p < 0.05.

**Table 2:** Volume (mm^3^) of left and right hippocampal subfields.

Labels	ADD+	ADD-	pAD	CIND	aAD	NC	*ANCOVA* (F_5,478_)^*a*^
L-Fissure	149.88 ± 33.57	154.83 ± 34.00	159.41 ± 24.29	162.21 ± 29.12	172.10 ± 34.43	160.11 ± 27.90	F = 3.13, p = 8.59E-3
L-Sub	308.11 ± 66.61	347.25 ± 72.95	369.83 ± 63.23	401.40 ± 60.71	424.64 ± 49.35	417.85 ± 56.92	**F = 46.39, p = 6.44E-39**
L-PaS	46.09 ± 16.36	48.78 ± 17.24	55.16 ± 17.36	56.40 ± 13.78	61.55 ± 12.74	59.79 ± 12.68	**F = 11.57, p = 1.48E-10**
L-PrS	208.21 ± 47.41	237.46 ± 53.27	257.19 ± 48.99	272.08 ± 44.17	289.84 ± 33.58	287.52 ± 40.76	**F = 43.68, p = 5.82E-37**
L-ML	408.85 ± 77.50	449.87 ± 87.82	482.60 ± 66.31	522.99 ± 68.09	546.98 ± 63.55	539.50 ± 65.19	**F = 52.67, p = 2.52E-43**
L-Tail	367.24 ± 68.69	423.95 ± 78.52	418.37 ± 68.78	462.28 ± 68.43	477.84 ± 73.85	472.28 ± 68.34	**F = 29.73, p = 3.03E-26**
L-CA3	172.54 ± 31.82	178.19 ± 37.09	200.53 ± 29.85	210.59 ± 27.94	214.03 ± 41.08	213.70 ± 32.77	**F = 24.35, p = 8.15E-22**
L-Fimbria	59.14 ± 22.42	63.84 ± 25.77	69.97 ± 26.30	81.24 ± 25.53	85.99 ± 18.62	84.19 ± 24.14	**F = 14.60, p = 2.60E-13**
L-GCMLDG	229.96 ± 40.92	245.29 ± 47.92	263.17 ± 35.50	287.45 ± 36.80	296.35 ± 39.54	295.08 ± 36.86	**F = 44.33, p = 1.98E-37**
L-CA1	477.85 ± 92.31	511.09 ± 98.12	544.91 ± 72.45	583.79 ± 74.48	614.49 ± 81.20	598.89 ± 75.10	**F = 34.57, p = 4.39E-30**
L-HATA	46.82 ± 11.96	46.62 ± 10.91	53.89 ± 9.95	57.78 ± 9.66	63.29 ± 10.32	60.46 ± 10.89	**F = 25.40, p = 1.07E-22**
L-CA4	202.21 ± 35.73	215.96 ± 42.23	231.55 ± 29.38	251.07 ± 30.58	259.05 ± 35.00	257.03 ± 30.88	**F = 43.16, p = 1.41E-36**
R-Fissure	181.48 ± 38.14	172.64 ± 33.38	178.40 ± 34.44	177.75 ± 30.78	188.08 ± 29.16	178.30 ± 33.36	F = 0.93, p = 0.45
R-Sub	329.74 ± 68.34	365.66 ± 73.24	390.26 ± 62.43	414.59 ± 61.67	441.39 ± 44.07	432.65 ± 56.53	**F = 39.00, p = 1.74E-33**
R-PaS	43.96 ± 15.75	47.85 ± 12.89	52.31 ± 14.01	54.45 ± 13.15	60.63 ± 12.27	56.68 ± 12.72	**F = 12.41, p = 2.51E-11**
R-PrS	217.71 ± 46.03	238.89 ± 40.17	258.08 ± 47.01	268.26 ± 39.84	287.46 ± 29.52	282.33 ± 38.26	**F = 33.00, p = 7.43E-29**
R-ML	449.71 ± 79.22	481.45 ± 81.13	516.56 ± 69.68	550.98 ± 72.96	575.95 ± 61.62	567.40 ± 69.10	**F = 39.56, p = 6.63E-34**
R-Tail	413.57 ± 73.19	462.34 ± 84.05	461.81 ± 73.33	498.45 ± 70.93	513.29 ± 74.27	505.78 ± 68.02	**F = 21.06, p = 5.16E-19**
R-CA3	194.06 ± 30.42	196.48 ± 36.07	210.36 ± 31.59	225.64 ± 32.92	231.05 ± 37.00	227.71 ± 34.42	**F = 16.61, p = 4.06E-15**
R-Fimbria	50.77 ± 19.31	55.49 ± 20.87	64.02 ± 22.51	68.86 ± 23.97	74.23 ± 18.37	73.72 ± 23.69	**F = 13.50, p = 2.55E-12**
R-GCMLDG	252.92 ± 38.89	261.94 ± 45.51	281.64 ± 38.63	302.33 ± 40.69	311.51 ± 41.24	307.42 ± 40.85	**F = 28.88, p = 1.47E-25**
R-CA1	533.81 ± 95.01	555.63 ± 90.33	592.31 ± 77.94	627.89 ± 81.62	655.64 ± 75.23	641.90 ± 81.02	**F = 25.74, p = 5.68E-23**
R-HATA	47.14 ± 9.79	49.75 ± 9.54	54.20 ± 8.87	56.58 ± 9.04	61.07 ± 7.45	58.96 ± 9.53	**F = 21.61, p = 1.74E-19**
R-CA4	222.69 ± 33.48	231.73 ± 39.16	246.65 ± 31.58	264.53 ± 33.82	272.52 ± 35.67	268.15 ± 33.52	**F = 29.13, p = 9.25E-26**

Values are expressed as mean ± SD.

Bold characters indicate significant results.

*Key*: L-, left; R-, right; ANCOVA, analysis of covariance.

^a^ANCOVA followed by Bonferroni correction was carried out to test the differences among groups (adjusting for the covariates age, gender, level of education, and total intracranial volume. Adjustment for multiple comparisons: p = 0.05/ 24 structures/ 6 groups = 3.47E-4). When the ANCOVA was significant, pairwise Bonferroni post hoc was applied. Whole hippocampus data is not shown.

**Table 3:** Bonferroni pairwise post hoc analysis.

Labels	NC vs. aAD	NC vs. pAD	NC vs. ADD+	NC vs. CIND	NC vs. ADD-	aAD vs. pAD	aAD vs. ADD+	aAD vs. CIND	aAD vs. ADD-	pAD vs. ADD+	pAD vs. CIND	pAD vs. ADD-	CIND vs. ADD+	CIND vs. ADD-	ADD+ vs. ADD-
L-Sub															
L-PaS															
L-PrS															
L-ML															
L-Tail															
L-CA3															
L-Fimbria															
L-GCMLDG															
L-CA1															
L-HATA															
L-CA4															
R-Sub															
R-PaS															
R-PrS															
R-ML															
R-Tail															
R-CA3															
R-Fimbria															
R-GCMLDG															
R-CA1															
R-HATA															
R-CA4															

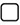
Statistically significant            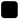
Statistically insignificant

Values are expressed as pairwise comparison P-values.

Bold characters with p < 0.05 indicate significant results.

*Key*: NA, not applicable; L-, left; R-, right.

Comparisons in each column are made based on both cognitive categories and the amyloid-PET statuses

**Table 4:** Comparison of hemispheric differences using paired t-tests.

Labels	NC	aAD	CIND	pAD	ADD-	ADD+
PaS	**t = 3.09,**	t = 0.34,	t = 1.55,	t = 1.21,	t = 0.39,	t = 0.90,
	**p = 2.26E-3**	p = 0.73	p = 0.12	p = 0.23	p = 0.69	p = 0.36
PrS	**t = 2.24,**	t = 0.44,	t = 1.61,	t = -0.18,	t = -0.22,	t = -1.88,
	**p = 0.02**	p = 0.65	p = 0.10	p = 0.85	p = 0.82	p = 0.06
Fimbria	**t = 7.42,**	**t = 4.14,**	**t = 7.27,**	t = 1.64,	**t = 2.23,**	**t = 4.01,**
	**p = 3.67E-12**	**p = 2.22E-4**	**p = 4.23E-11**	p = 0.11	**p = 0.03**	**p = 1.47E-4**
HATA	**t = 2.27,**	t = 1.38,	t = 1.88,	t = -0.22,	**t = -2.70,**	t = -0.22,
	**p = 0.02**	p = 0.17	p = 0.06	p = 0.82	**p = 0.01**	p = 0.81
Fissure	**t = -9.79,**	**t = -4.29,**	**t = -6.91,**	**t = -4.31,**	**t = -5.72,**	**t = -8.41,**
	**p = 1.27E-18**	**p = 1.45E-4**	**p = 2.59E-10**	**p = 1.38E-4**	**p = 3.00E-6**	**p = 3.50E-12**
Sub	**t = -5.38,**	**t = -2.91,**	**t = -4.99,**	**t = -3.51,**	**t = -2.53,**	**t = -3.79,**
	**p = 2.14E-7**	**p = 6.37E-3**	**p = 2.00E-6**	**p = 1.28E-3**	**p = 0.01**	**p = 3.15E-4**
ML	**t = -9.13,**	**t = -4.61,**	**t = -8.13,**	**t = -5.08,**	**t = -3.77,**	**t = -5.87,**
	**p = 9.32E-17**	**p = 5.60E-5**	**p = 5.05E-13**	**p = 1.50E-4**	**p = 7.28E-4**	**p = 1.33E-7**
Tail	**t = -9.18,**	**t = -4.97,**	**t = -9.57,**	**t = -5.95,**	**t = -3.87,**	**t = -8.81,**
	**p = 7.11E-17**	**p = 2.00E-5**	**p = 2.18E-16**	**p = 1.00E-6**	**p = 5.58E-4**	**p = 6.59E-13**
CA3	**t = -7.85,**	**t = -4.56,**	**t = -7.30,**	**t = -2.48,**	**t = -4.14,**	**t = -7.26,**
	**p = 2.88E-13**	**p = 6.60E-5**	**p = 3.61E-11**	**p = 0.01**	**p = 2.73E-4**	**p = 4.31E-10**
GCMLDG	**t = -6.91,**	**t = -3.71,**	**t = -7.38,**	**t = -4.86,**	**t = -3.11,**	**t = -6.85,**
	**p = 6.95E-11**	**p = 7.49E-4**	**p = 2.41E-11**	**p = 2.70E-5**	**p = 4.10E-3**	**p = 2.46E-9**
CA1	**t = -10.85,**	**t = -6.24,**	**t = -10.23,**	**t = -5.74,**	**t = -4.43,**	**t = -6.34,**
	**p = 1.06E-21**	**p = 4.67E-7**	**p = 5.96E-18**	**p = 2.00E-6**	**p = 1.20E-4**	**p = 1.96E-8**
CA4	**t = -7.09,**	**t = -4.10,**	**t = -7.73,**	**t = -4.72,**	**t = -3.37,**	**t = -6.76,**
	**p = 2.47E-11**	**p = 2.50E-4**	**p = 3.99E-12**	**p = 4.10E-5**	**p = 2.10E-3**	**p = 3.55E-9**

Values are expressed as t-statistics and respective P-values.

Bold characters with p < 0.05 indicate significant results.

## Results

### Demographics

Table 1 shows no significant differences in terms of age (F_5, 478_ = 1.29, p = 0.26) and gender (*χ*^*2*^ test: *χ*^*2*^ = 6.60, p = 0.25) were observed among the groups. Levels of education (F_5, 478_ = 2.54, p = 0.02) were significantly different when all the groups were compared, and an additional pairwise comparison revealed that the difference was only specific to CIND and ADD+ groups (post hoc: CIND versus ADD+, p = 0.01). The MMSE scores were significantly different between the groups (F_5, 478_ = 73.44, p = 1.08E-56). However, the post hoc analyses showed that the scores were not different between aAD and NC, pAD and CIND, and ADD- and ADD+ groups.

### Group comparisons and hemispheric asymmetry findings

As summarized in Tables 2 and 3, comparisons of the six groups showed that hippocampal fissure volume was not significantly different between them (after adjusting for Bonferroni correction, p = 0.05/ 24 structures/ 6 groups = 3.47E-4). Additionally, the bilateral PaS, left CA3, and right fimbria did not show any significant volume differences between the aAD and pAD groups. The left fimbria and right CA3 showed significant atrophy in the supposed early AD progression stages, though they did not show any significant atrophy difference between the pAD and ADD+ groups. The percentile volume loss was higher in the prodromal AD to AD dementia stage than in the preclinical AD to prodromal AD stage. Predominantly, there was a higher percentile volume loss in the left hemisphere than in the right hemisphere.

### Findings in NC and aAD groups

**Fig 1:**
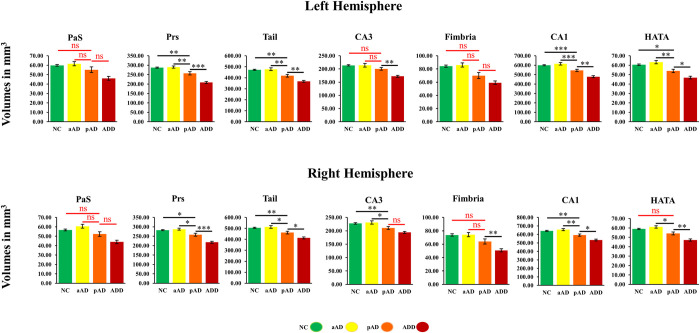
Hippocampal subfield volumes in NC, aAD, pAD, and ADD groups adjusting for the covariates age, sex, level of education, and intracranial volume. Error bars indicate two standard errors. There were seven prominent regions among the entire 13 regions. NC, normal controls; aAD, asymptomatic Alzheimer Disease; pAD, prodromal Alzheimer Disease; ADD, Alzheimer Disease Dementia; n.s, not statistically significant; *, p < 0.05; **, p < 0.01; ***, p < 0.001.

Subfield volumes between NC and aAD groups were not significantly different from each other (Tables 2 and 3; Fig 1). We observed left-right hemispheric differences in both groups, with significantly larger right hemisphere volumes than in the left hemisphere, except in the PrS, PaS, fimbria, and HATA (Table 4). In these regions, the volumes on the right were smaller than those on the left. However, in the aAD group, these differences were not statistically significant, except in the fimbria. In addition, the asymmetries were higher in the NC group than in the aAD group. Compared to other subfields, the left-right PaS volumes in NC and aAD groups were weakly correlated (Table 5).

**Table 5:** Correlations between the two hemispheres.

Labels	NC	aAD	CIND	pAD	ADD-	ADD+
Fissure	**r = 0.66, p = 1.90E-25**	**r = 0.77, p = 5.55E-8**	**r = 0.66, p = 1.17E-16**	**r = 0.66, p = 1.60E-5**	**r = 0.87, p = 3.31E-10**	**r = 0.62, p = 8.66E-9**
Sub	**r = 0.77, p = 1.21E-39**	**r = 0.74, p = 3.66E-7**	**r = 0.89, p = 1.92E-41**	**r = 0.85, p = 1.22E-10**	**r = 0.85, p = 2.30E-9**	**r = 0.75, p = 7.41E-14**
PaS	**r = 0.40, p = 8.37E-9**	r = 0.21, p = 0.21	**r = 0.49, p = 1.34E-8**	**r = 0.64, p = 4.30E-5**	**r = 0.67, p = 4.70E-5**	**r = 0.25, p = 0.03**
PrS	**r = 0.67, p = 1.42E-26**	**r = 0.52, p = 1.54E-3**	**r = 0.81, p = 1.87E-29**	**r = 0.82, p = 2.74E-9**	**r = 0.76, p = 8.24E-7**	**r = 0.59, p = 6.36E-8**
ML	**r = 0.80, p = 1.32E-44**	**r = 0.82, p = 1.30E-9**	**r = 0.86, p = 5.90E-36**	**r = 0.83, p = 6.95E-10**	**r = 0.85, p = 1.61E-9**	**r = 0.72, p = 1.28E-12**
Tail	**r = 0.72, p = 1.37E-32**	**r = 0.84, p = 4.05E-10**	**r = 0.82, p = 8.10E-31**	**r = 0.82, p = 2.31E-9**	**r = 0.77, p = 3.84E-7**	**r = 0.80, p = 2.19E-17**
CA3	**r = 0.72, p = 3.21E-33**	**r = 0.84, p = 2.02E-10**	**r = 0.74, p = 8.04E-22**	**r = 0.71, p = 2.00E-6**	**r = 0.78, p = 3.42E-7**	**r = 0.68, p = 6.69E-11**
Fimbria	**r = 0.66, p = 4.72E-26**	**r = 0.60, p = 1.74E-4**	**r = 0.72, p = 2.24E-20**	**r = 0.63, p = 5.40E-5**	**r = 0.63, p = 1.79E-4**	**r = 0.65, p = 5.19E-10**
GCMLDG	**r = 0.80, p = 2.04E-44**	**r = 0.82, p = 1.61E-9**	**r = 0.84, p = 2.36E-33**	**r = 0.82, p = 1.98E-9**	**r = 0.80, p = 8.09E-8**	**r = 0.75, p = 4.69E-14**
CA1	**r = 0.75, p = 1.03E-36**	**r = 0.88, p = 5.38E-12**	**r = 0.82, p = 2.07E-30**	**r = 0.79, p = 1.59E-8**	**r = 0.83, p = 1.11E-8**	**r = 0.69, p = 3.76E-11**
HATA	**r = 0.60, p = 9.43E-21**	**r = 0.48, p = 3.63E-3**	**r = 0.73, p = 6.56E-21**	**r = 0.64, p = 3.50E-5**	**r = 0.81, p = 3.93E-8**	**r = 0.43, p = 1.80E-4**
CA4	**r = 0.77, p = 8.45E-40**	**r = 0.85, p = 1.42E-10**	**r = 0.83, p = 1.57E-31**	**r = 0.81, p = 4.12E-9**	**r = 0.80, p = 8.12E-8**	**r = 0.73, p = 4.90E-13**

Values are expressed as correlation coefficient values ‘*r*’ and respective P-values.

Bold characters with p < 0.05 indicate significant results.

### Findings in CIND and pAD groups

As shown in Tables 2 and 3 and Fig 1, bilateral volumes of the hippocampal tail, CA1, ML, GCMLDG, CA4, and right CA3 significantly differed between CIND and pAD groups, with larger volumes in the CIND group than in the pAD group. In addition, left-right hemispheric asymmetries were significantly higher in CIND than in the pAD group. All other subfields except PrS, PaS, HATA, and fimbria (Table 4) had larger right hemisphere volumes. The left-right PaS volumes in CIND and pAD groups were moderately correlated (Table 5).

### Findings in ADD- and ADD+ groups

The ADD- group had significantly larger volumes than the ADD+ group in the bilateral tail, SUB, PrS, and left ML (Tables 2 and 3; Fig 1). In addition, subfields other than the PrS, PaS, HATA, and fimbria (Table 4) had larger right hemisphere volumes than left hemisphere volumes. Inconsistent with the between NC and aAD and the between CIND and pAD group findings, the left-right hemispheric asymmetry was lower in the ADD- group than in the ADD+ group. In addition, the PaS left-right hemispheric volumes were weakly correlated in the ADD+ group and moderately correlated in ADD- group (Table 5).

## Discussion

In this study, we tried to improve the diagnosis of AD by not taking the traditional focus on the total hippocampus but by instead focusing on hippocampal subfield volume. To the best of our knowledge, the current study is the first large-scale study of the Korean population to investigate changes in distinct hippocampal subfield volumes that accompany the Aβ burden and cognitive changes in AD and stages preceding AD. As expected, 1) there were asymmetries in the subfield volumes among the groups that were distinct for the respective group, 2) the Aβ negative group had larger subfield volumes than the Aβ positive group in both the mild cognitively impaired and the severe cognitively impaired groups, and 3) the subfield volumes showed a decreasing trend along the AD continuum. The additional findings from the study were that (1) there was an intense and accelerated focal atrophy at the pAD, which was specific to prodromal AD subjects, (2) certain hippocampal subfields showed no volume loss till the late prodromal AD or very early ADD stage, and (3) the subfield volume loss in ADD might be stronger than in other suspected non-Alzheimer’s pathology. Most of the findings are in the expected directions, which could be taken to mean that the information might be used to improve the diagnosis of Alzheimer’s disease.

### Findings of the study

Neurodegeneration is believed to cause cognitive dysfunction, which is expected to be mediated by the deposition of β-amyloid [20]. Approximately 10–30% of cognitively normal individuals have abnormal levels of β-amyloid, suggesting a biological relevance to normal aging [41, 42] and also suggesting that the abnormal levels of β-amyloid are not sufficient to cause clear cognitive symptoms [6]. Prior studies show that β-amyloid positive cognitively normal, mild cognitively impaired, and AD dementia subjects at baseline showed greater cognitive and global deterioration compared to β-amyloid negative subjects [43–45]. Without neurodegeneration, which provides vital pathological staging, the differences between the plaques and tangles cannot be formally captured. Furthermore, standalone PET imaging cannot function as a significant prediction tool for future cognitive impairment without the use of MRI [46].

### Differential volume loss among groups

The bilateral comparison of the six diagnostic groups showed that all the subfields volumes differed between the groups, except for the hippocampal fissure. However, when the diagnostic group volumes were compared using pairwise comparison, certain subfield volumes between a few groups were not different.

### Comparison between NC and aAD

In line with previous studies [47, 48], there were no significant differences in volumes between the NC and aAD groups. Although, a few studies have shown marginal statistical significance in volume differences of one or two subfields between aAD and NC, with aAD showing lower volumes than the NC [49, 50]. These studies have failed to find clear differences in the other subfields they studied. The differences reported could be due to the sampling and sample size discrepancies, procedural variations, and most notably, ethnic variability. We observed a trend level increase in the subfield volumes of the aAD group compared to the NC group, which is similar to the observations made in the final model of temporal ordering of biomarkers in the Dominantly Inherited Alzheimer Network study [51].

No clear differences in the normal aging-associated hippocampal atrophy rates have been observed between the normal control and the MCI subjects with the same β-amyloid status [52], and an accelerated volume loss was seen specifically in the MCI group [53–55]. Thus, the current stage might be too early to see a clear volume loss, and a longitudinal study between the NC and aAD groups might provide a much clearer picture of the underlying mechanisms.

### Comparisons between cognitively normal and mild cognitively impaired

It is shown that hippocampal atrophy can act as a strong predictor of AD progression and can discriminate the MCI from the cognitively normal [56]. When we compared the NC and aAD groups to the CIND group bilaterally, we observed no clear differences in the subfield volumes. Interestingly, when we compared the NC and aAD groups to the pAD group bilaterally, most of the subfield volumes showed clear differences; this indicates that the pAD is the transitional stage in the AD continuum, and the differences in the subfield volumes might be useful to classify subjects with mild cognitive impairment into pAD and CIND groups even before the β-amyloid imaging. A more extensive study might help clarify the underlying mechanisms of this transitional stage.

### Comparisons between cognitively normal and severely impaired

In line with previous studies [25, 57, 58], comparing the NC and aAD groups with the ADD+ and ADD- groups yielded very clear differences in all the subfields, clearly differentiating the normal controls from subjects with dementia.

### Comparisons between pAD and CIND

In line with an earlier study [59], in the MCI groups pAD and CIND, we observed larger subfield volumes in the CIND group than in the pAD group. Additionally, we observed no significant bilateral volume difference of the subicular complex (SUB, PrS, and PaS), but there was atrophy in the bilateral tail, CA1, ML, GCMLDG, CA4, and right CA3.

### Comparisons between mild cognitively impaired and severely impaired

We then compared the pAD & CIND groups with the ADD+ & ADD- groups. There were clear differences when the CIND group was compared with the ADD+ and ADD- groups. The CIND might be a result of cognitive impairment resulting from the normal aging process, metabolic disturbance, substance abuse, or head trauma [28, 60]. pAD & ADD+ groups showed clear differences in all subfields, except the bilateral PaS, left fimbria, and right CA3, suggesting that these regions are atrophied only in the late AD stages, located some distance from the suspected pathological initiation sites, the CA1 and the subiculum [61–63].

### Comparisons between ADD+ and ADD-

Finally, a comparison of ADD+ and ADD- groups showed differences in the bilateral SUB, PrS, tail, and left ML, suggesting that the volume atrophy in ADD+ might be more severe than in ADD- or other types of dementia [64]. All other subfields showed no clear differences. In addition, several subjects in the ADD- group has been shown to demonstrate Aβ positivity in a longitudinal study [65].

### Comparisons between CIND and ADD-

Individuals in the group CIND or Aβ- MCI mostly have less cognitive and functional impairment at baseline than patients with pAD or Aβ+ MCI and individuals in the group Aβ- MCI are less likely to convert to AD. In Aβ- MCI, the simultaneous presence of several comorbidities makes it difficult to pinpoint the cause of the cognitive symptoms. In ADD- or Aβ- ADD individuals, non-AD etiologies like subclinical depression and vascular abnormalities may account for AD-like phenotype. However, individuals with ADD+ or Aβ+ ADD and Aβ- ADD do not have very strong differences in cognitive impairments [66].

### The trajectory of volume loss

Based on the results, we expect that the trajectory of AD progression might be from NC to aAD to pAD to ADD+. In cognitively unimpaired and MCI subjects, those diagnosed with Aβ-negativity showed a higher hemispheric volume difference than those diagnosed with Aβ-positivity. In contrast, in severely cognitively impaired subjects, those diagnosed with Aβ-positivity showed a higher hemispheric volumetric difference than those diagnosed with Aβ-negativity [64]. Our study shows the importance of separating the Aβ positive subjects from the Aβ negative ones.

### Selective vulnerability among subfields

Neuropathological studies have reported that the AD continuum reflects a complex and ordered sequential process with the atrophy beginning at the anterior CA1-subiculum regions and progressing toward other subfields [61, 62, 67, 68]. Also, another study has reported presubicular-subicular complex atrophy in the earliest stages of AD [69]. In line with the previous findings, we observed atrophy in both the CA1 and subiculum regions in a comparison between the cognitively unimpaired groups and the pAD group. Additionally, atrophy in other subfields was observed in a comparison between the cognitively unimpaired groups and the pAD group. The bilateral parasubiculum, left CA3, and right fimbria situated at some distance from the CA1-subiculum regions show no atrophy until the late AD stages. The parasubiculum is a transitional area sandwiched between the presubiculum and the entorhinal area [19] and is postulated to play a vital role in spatial navigation and the integration of head-directional information [70]. The fimbria extends to the fornix, the brain’s white matter, and the CA3 is expected to be the largest subregion in the hippocampus [71]. The atrophy of the parasubiculum and CA3 might suggest terminal disease propagation stages that later spread to other brain regions.

### Hemispherical asymmetry in subfield volume loss

In line with an earlier study [72], we observed left-right hemispheric asymmetry in the atrophy pattern. In most subfields, the left hemispheric volumes showed more severe atrophy than the right hemispheres. The early atrophy of the left fimbria and the right CA3 was observed but not the right fimbria and the left CA3, which show a hemispherical difference in the atrophy patterns of these two regions [73]. These hemispheric volume differences are seen in almost all subfields, with larger right hemisphere volumes except in the PrS, PaS, and HATA.

## Limitations

The first limitation of the present study is that it has a cross-sectional design. A future longitudinal study would offer a more convincing way to test our hypotheses.

Due to the difficulty of obtaining amyloid-PET data, data from cognitive tests, and MRI data on the same participants, the number of participants in the different groups is unbalanced. Moreover, in the cognitively unimpaired and mild cognitively impaired groups, the number of amyloid-negative subjects is larger than the number of amyloid-positive subjects, but in the severely cognitively impaired group, the number of amyloid-positive subjects is larger than the number of amyloid-negative subjects. These unequal sample sizes decrease the statistical power of the analyses. Finally, the unequal sample sizes combined with substantial differences in the variances’ values increase the number of type-I errors for post hoc tests when comparing groups.

A clear limitation of the present study is that the MRI scans’ resolution is lower than those obtained with newer equipment. Imperfect measures are ubiquitous in biomedical research, and statisticians have dealt with this issue in detail [74]. A common conclusion is that having imperfect measures underestimates the true relationships. In the present case, repeating the present analyses using comparable datasets with MRI scans of higher resolution would most likely lead to substantially increased values of *r*s and *d*s in many cases. So, most likely, many of the conclusions would become stronger.

Finally, although a single-subject analysis would give a deeper understanding of the obtained results and their significance in clinical practice, we limit ourselves to the group-level analysis of the experimental groups.

## Conclusions

We conclude that the results suggest that early depositions of amyloid in cognitive normal stages are not accompanied by significant bilateral subfield volume atrophy. Early subfield volume atrophy associated with the β-amyloid burden may be characterized by more symmetrical atrophy in CA regions than in other subfields. So, hippocampal subfield volumetry shows promise in improving the diagnosis of Alzheimer’s disease. Clearly, more research is needed.

## Supporting information

S1 Fig An illustration of neuro-anatomically distinct non-homogenous hippocampal subfields as segmented by the contemporary method used in the study. CA–cornu ammonis, GCMLDG–granule cell layer of the dentate gyrus, HATA–hippocampus-amygdala-transition area.
(DOCX)

S2 Fig Distribution of study subjects with respect to age against Mini-Mental State Examination score. CIND, cognitive impairments that are not dementia.
(DOCX)

S1 Table Hippocampal subfield regions and their major functions. (DOCX)

S2 Table Classification of the study participants. (DOCX)

S1 File (XLSX)
