# Supplementary figures and images for: Can hippocampal subfield measures supply information that could be used to improve the diagnosis of Alzheimer’s disease?
Source: PLoS One. 2022 Nov 3;17(11):e0275233. doi: 10.1371/journal.pone.0275233 (PMC9632892; doi:10.1371/journal.pone.0275233)

**
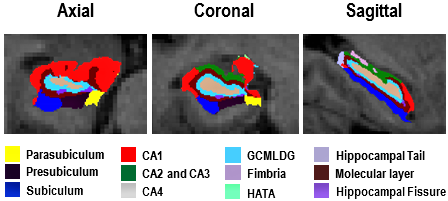
**

Supplement: S1 Fig — CA–cornu ammonis, GCMLDG–granule cell layer of the dentate gyrus, HATA–hippocampus-amygdala-transition area. (DOCX) [file pone.0275233.s001.docx]

**
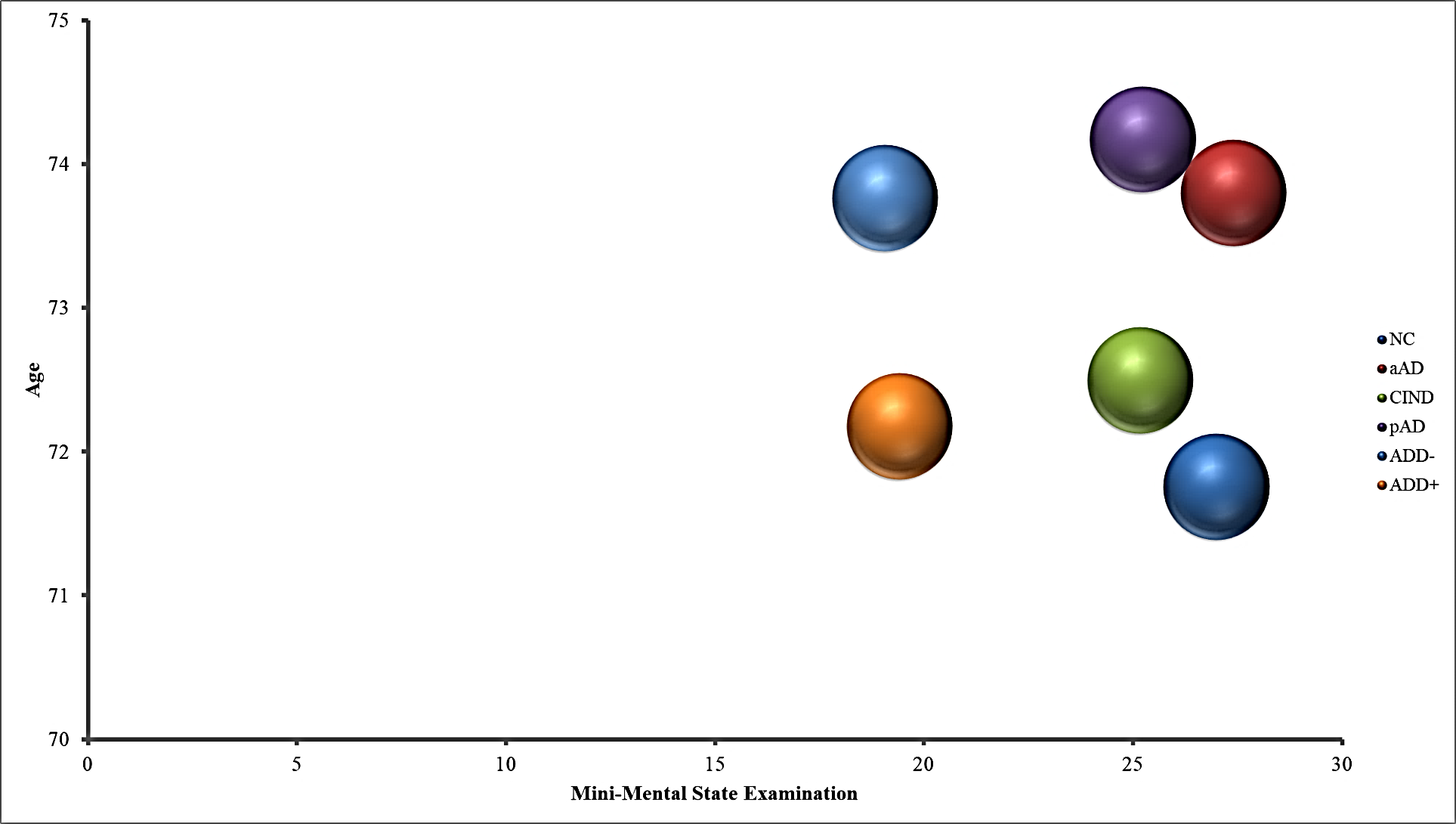
**

Supplement: S2 Fig — CIND, cognitive impairments that are not dementia. (DOCX) [file pone.0275233.s002.docx]
