# Supplementary material for: Can hippocampal subfield measures supply information that could be used to improve the diagnosis of Alzheimer’s disease?
Source: PLoS One. 2022 Nov 3;17(11):e0275233. doi: 10.1371/journal.pone.0275233 (PMC9632892; doi:10.1371/journal.pone.0275233)
Supplement: S1 Table — (DOCX) [file pone.0275233.s003.docx]

**Supplementary** **table S2. Classification of the study participants**

|  | Cognitively unimpaired | Mild cognitive impairment | Severe cognitive impairment |
| --- | --- | --- | --- |
| β-amyloid positive | Asymptomatic Alzheimer’s disease  (aAD, n=34) | Prodromal AD  (pAD, n=34) | AD dementia positive  (ADD+, n=70) |
| β-amyloid negative | Normal control  (NC, n=192) | Cognitive impairments that are not dementia  (CIND, n=118) | AD dementia negative  (ADD-, n=30) |
