# Supplementary material for: Can hippocampal subfield measures supply information that could be used to improve the diagnosis of Alzheimer’s disease?
Source: PLoS One. 2022 Nov 3;17(11):e0275233. doi: 10.1371/journal.pone.0275233 (PMC9632892; doi:10.1371/journal.pone.0275233)
Supplement: S2 Table — (DOCX) [file pone.0275233.s004.docx]

**Supplementary table S1. Hippocampal subfield regions and their major functions**

| **Label** | **Functions** | **Reference** |
| --- | --- | --- |
|  |  |  |
| Fissure | Neurogenic niche and possible dispersion of neuroprogenitors from subgranular niches to CA fields | (Zhang et al., 2014) |
| Sub | Temporal control of reinforced behavior | (O'Mara et al., 2009) |
| PaS | Representation of location in the environment | (Taube, 1995) |
| PrS | Head direction information | (Robertson et al., 1999) |
| ML | Gateway for sensory information into hippocampus | (Sancho-Bielsa et al., 2012) |
| Tail | Storage of spatial representation of the environment | (Maguire et al., 2000) |
| CA3 | Rapid encoding of new spatial information within short-term memory, pattern completion | (Kesner, 2007; Neunuebel and Knierim, 2014) |
| Fimbria | Spatial learning and navigation, memory | (Sutherland and Rodriguez, 1989) |
| GCMLDG | Pattern separation | (Neunuebel and Knierim, 2014; Yassa and Stark, 2011) |
| CA1 | Retrieval of remote episodic memory and important for autonoetic consciousness | (Bartsch et al., 2011) |
| HATA | Contextual fear learning and emotional memory processes | (Fudge et al., 2012) |
| CA4 | Regulation of stress | (Elvsåshagen et al., 2016; Hayes et al., 2017) |

Sub, subiculum; PaS, parasubiculum; PrS, presubiculum; ML, molecular layer; Tail, hippocampal tail; CA, cornu ammonis; GCMLDG, granule cell layer of dentate gyrus; HATA, hippocampus-amygdala-transition area.
